# New Frontiers or a Bursting Bubble? Psychedelic Therapy Beyond the Dichotomy

**DOI:** 10.3389/fpsyt.2021.727050

**Published:** 2021-09-10

**Authors:** Tehseen Noorani, Jonny Martell

**Affiliations:** ^1^Department of Anthropology, Durham University, Durham, United Kingdom; ^2^Centre for Psychedelic Research, Imperial College London, London, United Kingdom

**Keywords:** psychedelic therapy, bubble, opportunity, psychedelics, hallucinogens, regulation

## Introduction

The publication in April 2021 of the Imperial College London Phase II study investigating the efficacy of psilocybin-assisted therapy vs. escitalopram for depression reported differences in the primary outcome measure (the QIDS-SR16) between experimental and control arms as statistically insignificant (1). However, secondary measures of depression, and other relevant measures (see Appendix), favored psilocybin to escitalopram. Soon thereafter, a range of expert commentaries offered interpretations, including that the researchers were unfortunate in their choice of pre-registered primary outcome, the trial was underpowered perhaps revealing an overconfidence in designing the study, and the limitations of depression rating measures to capture the return of positive mood and well-being.

Partially in response to these publications, discussions in research and online communities have grown around the over-hyping of psychedelic treatments, bringing into focus concerns over psychedelic therapy trial methodology [for a peer-reviewed critique, see (2)]. This opinion piece explores these concerns to propose a response to this special issue's question, “Can Psychedelic Therapies Open a New Frontier in Mental Healthcare (Or Will the Bubble Burst)?” Drawing on our experiences of working within psychedelic clinical trials and NHS psychiatry, we offer here a deflationary answer to this question, suggesting both will occur and outlining some of the facets, stakes and opportunities entailed.

## Part I: The Bursting Bubble

The enthusiastic reception and excitement over the potential of psychedelics in academic, medical, public, and even political arenas is buttressed by the growing call for new psychiatric treatments, concerns over the long-term prescription of antidepressants to growing patient numbers without a concomitant reduction in psychiatric morbidity, and increasing evidence of withdrawal effects (3, 4). We define the “psychedelic therapy bubble” as an overestimation of its promise that is not justified by what is and can be known about its therapeutic potential. Coining the term promissory science, Davis and Abraham describe how,

“in connection with the manufacturer's promotion of apparently hopeful clinical trial results in the lead up to a new drug application for marketing approval.both the hopeful results and the novelty of the new drug's hypothesized mechanism of therapeutic efficacy may be elevated to great clinical significance to an expectant medical profession, patient population and stock market” [(5), p.268] [see also (6, 7)].

Understanding the psychedelic therapy bubble in terms of promissory science highlights the feedback loops whereby inflated expectations are themselves shaping psychedelic therapy's potential (8). Economically, the bubble sustains sufficient initial investments to offset the direct and infrastructural costs of bringing psychedelic therapies to market. Consequently, commercial imperatives of efficiency, standardization, and scalability are steering ongoing research. Advocates of these processes argue that this is either appropriate or at least necessary, in order to re-invigorate psychiatry's tired pharmacopeia (9). Culturally, the success of psychedelic therapy has been steered by actors seeking redemption for psychedelic-assisted and related healing practices in the wake of psychedelic prohibition at the end of the 1960s, and the researchers among them have brought particular interpretive frameworks for understanding the nature and value of psychedelic experiences. This includes the foregrounding of the therapeutic value of the mystical-type experience, which has begun to be problematized from various quarters as overly simplistic [e.g., (10)].

The concern with psychedelic therapy being a bubble that may burst is sometimes reduced to a warning against a repeat of drug prohibitionist laws and policies [e.g., (11)]. Yet the more colorful narrative of psychedelic research as a casualty in Nixon's fight against the counterculture has in recent years been tempered by historical investigation into the effects of an impasse between paradigms for conducting psychedelic therapy research and the growing imperatives of standardized randomized controlled trial (RCT) designs (12, 13). Moreover, today's situation is different in key ways: firstly, scholars have documented how medical professionals, regulators, and patient groups have pushed for “pragmatic” and “adaptive” trial designs in the intervening decades (14). Secondly, today's psychedelic clinical researchers are self-consciously aware of, and keen to prevent, a return to psychedelic prohibition, carefully proffering a “sober objectivity,” and emphasizing psychedelics as therapeutic agents as opposed to subversive agents of social and political change (15). Thirdly, we suggest psychedelic therapy is well-suited to the growing calls for psychiatry to be more oriented to the relational (16).

Nevertheless, bubbles burst, and we offer four axes along which to consider this in relation to psychedelic therapies. Firstly, critiques of psychedelic therapy trial publications have warned of common features of the RCTs that are likely to be inflating effect sizes: participant self-selection, stringent screening procedures, small sample sizes, and difficulty in maintaining blinding in research trials (2). Secondly, as the therapies gain legitimacy, the psychedelic research community is itself changing. Until recently, this community has comprised researchers who were willing to accept the professional ramifications of working in a stigmatized area. While the early advocates of any new treatment can be expected to be enthusiastic, the interaction effects of any researcher bias with psychedelics' sensitivity to the context of their use would contribute especially large outcome confounders, leading to limited replication of the early findings. Thirdly, we should anticipate a growth in adverse outcomes as the hype grows, the participant/patient pool is widened, and psychedelic therapies are provided in more streamlined ways. Increasingly diverse patient populations hopeful of being cured will experience rocky “landings” post-treatment, the cost of which will be borne downstream of trial analysis end points, thereby falsely inflating favorable health economic calculations. And fourthly, we are yet to understand the nature and extent of ethical dilemmas that will emerge. For example, with regards to “false memory syndrome,” the complexity of working with the veracity of unmediated knowledge has recently been explored (17), posing a challenge for manualized and protocolized therapy training programs.

## Part II: New Frontiers

Recognizing that psychedelic therapy is a bubble that will burst should not be taken to mean that psychedelics do not offer “new frontiers.” On the contrary, we outline four areas below in which psychedelic therapy has the potential to transform mental healthcare, even if it is not approved and mainstreamed to the extent currently anticipated.

### Novel Compounds

Psychedelic research has stimulated interest in a trove of new psychedelic-inspired compounds (for example, see MagicMed Industries Inc's Psybrary™), with differing pharmacological and risk profiles. We can expect the effects they produce to range from therapeutic to toxic and psychoactive to non-psychoactive. With the proliferation of these compounds, we anticipate a growing discussion about the necessity of the psychedelic experience for therapeutic gain [e.g., (18, 19)]. Additionally, the development of “neutralization technologies” for terminating the psychedelic experience and drug administration through novel target-controlled intravenous infusion techniques (20) suggest to us that therapeutic practices will be developed that combine both “shutting down” and “opening up” to offer new options for experiential manipulation, in line with the growing call for personalized medicine.

### Psychedelic Therapy as Driving Interest in Suggestion

Psychedelic therapy trials are generating data we suggest could fruitfully be conceptualized through the framework of the sensitivity of therapeutic altered states to suggestion. This connects several literatures: a tradition of psychedelic research that proposes the drugs produce states of heightened suggestibility against a longer historical interest in hypnosis (21–24); psychedelics as psychoplastogens, increasing cognitive and emotional flexibility (25–27)); the field of placebo studies (28–30); ritual theory (31, 32) and the study of “common factors” across psychotherapies (33) alongside other process-oriented research into psychological change. By bringing these literatures into a fuller encounter, we imagine a range of conceptual distinctions, methodological considerations, and theories, animating new directions for psychedelic therapy research.

Considering the above literatures, it would be timely to bring together psychedelic researchers and experts in these related areas, to identify trial designs and interpretive frameworks that support mutually fruitful lines of inquiry. Interdisciplinary research could reveal the processes by which the psychedelic experience may be “baking in” a particular message or self-reorientation that has been primed or reinforced by the other study components. Without wanting to foreclose what such conversations will open up, we are particularly interested in two avenues of inquiry: firstly, more fully measuring the expectations held by participants at different stages of the intervention would reveal more than potential confounds within trials—it could teach us about the looping effects of the bubble itself, by indexing how trials are themselves differentially emplaced within a timeline of the evolving hype around psychedelic therapies. Secondly, a fuller engagement with placebo studies suggests the need to consider more fully the role of uncertainty, hesitation, and doubt in healing through psychedelic therapy (31, 34), and how these are cultivated through drug-set-setting configurations that offer the opportunity to “[traffic] in human possibilities rather than in settled certainties” (35). Reflections on the history of placebo studies may also caution against reprising politically and ethically fraught attempts to identify the suggestible “type” of person (Phoebe Friesen, personal communication). We worry, however, that such interdisciplinary enquiry is being sidelined in the rush to approve particular psychedelic therapy packages for the treatment of particular indications.

### The Role of Care

A broad intersecting of psychedelic research and related research literatures also suggests a deeper valuing of the role of care structures in psychiatric care writ-large. While the imperative in placebo-controlled antidepressant trials has been to minimize the placebo response by limiting patient expectancy and therapeutic contact (36, 37), this is inverted in the enriched spaces and care protocols developed for psychedelic therapies (38). These spaces and protocols challenge the reductionism of psychopharmacological interventions by drawing attention to the relational aspects of care, moving from a compartmentalized focus on symptoms in need of treatment to a more holistic view of human suffering and the value of meaning-making in health and wellness. More broadly, the rise of the psychedelic therapy bubble has contributed to the growing awareness of a wider ecology of healing practices that center the body, space-making, and spirituality.

However, the significant opportunities for psychedelic researchers to refocus psychiatric treatments on enriching and optimizing relations of care is compromised by industry pressures, including commercial incentives to foreground the “drug treatment” model over the crucial (39) but costly psychotherapeutic work, and a regulatory system designed to evaluate the administration of drugs as stand-alone treatment modalities. Emphasizing the relations of care that appear to us to be key to safe and effective psychedelic therapy will also require coordination across scales and disciplines. For example, some psychedelic advocates are beginning to make the economic case for psychedelic therapies by appealing to the long-term cost-savings achieved if psychedelic therapies are considered a curative rather than a palliative care treatment [in relation to MDMA-assisted psychotherapy, see (40)]. It remains unclear whether this strategy will persuade healthcare payers.

### Regulatory Dynamism

Finally, we suggest that psychedelic therapy research heralds an opportunity to rethink the regulation of drug research more broadly, owing to how sensitive psychedelic experiences are to the context of their use. Some researchers have called for more detailed measurement of the context of psychedelic therapy in order to better grasp its therapeutic mechanisms [cf. (38, 41, 42)], something the growth in survey approaches is endeavoring to do [e.g., (43)]. We wonder what it might mean to circumscribe the generalizability of research findings much more narrowly, acknowledging not simply that there are additional contextual parameters that need taking into account but that it may not be possible to determine intervention effects in any general sense, given the plethora of “experimental contingencies” (2:8) in any given context.

The need for a more context-sensitive and systematic coupling of research pre- and post-approval is important. Psychedelic therapies seek approval based upon safety and efficacy data, and yet their very approval may affect their efficacy and safety profiles, as mediated by expectations and understandings of the substances borne of the evolving bubble that their hype is generating. Psychedelic therapies also challenge any easy separation of safety and efficacy, as when, for example, powerful new meanings and senses of purpose are generated that are therapeutic, but only with appropriate structures of support and care. Existing post-approval regulatory landscapes are poorly equipped to meet the needs of patients for up-to-date (re-)evaluations of safety and efficacy. Regulators lack comprehensive and timely data for determining post-approval safety issues, much less changes in efficacy, and have failed to enforce the surveillance measures they currently demand of the pharmaceutical industry (44). The shift in onus of responsibility to patients and providers to deliver on what is called “post-marketing surveillance” *via* reporting systems has largely failed to deliver, due to under-use and the constraints posed by imperatives for data anonymization (45).

One logical endpoint of the calls for greater context sensitivity and the ongoing monitoring of changing safety and efficacy profiles is in the technically-mediated surveillance of psychedelic therapy through adjunctive devices and apps, as seen in calls for the use of ever-more-granular monitoring technologies in order to adequately power research (46). It remains to be seen whether such “field approaches” can provide useful findings. Meanwhile, the normalization of such technologies raises substantial legal and political concerns, in particular around the case for reconsidering proprietary rights in relation to data capture.

## Discussion

In this opinion piece we have sought to foster greater critical discourse around the broader regulatory, cultural and economic forces shaping psychedelic research. While we have argued for the need to be attentive to how the psychedelic therapy bubble is shaping its potential, we end on a note of optimism. In considering the “bubble” as a public health measure in the wake of the Covid-19 pandemic, Appleton (47) invites us to imagine bubbles as affording a protected space in the knowledge that such a space is temporary. We might then consider this moment an opportunity to develop robust methodologies and alliances across research disciplines and with stakeholder groups, and to inquire into the most pressing research questions of psychedelic therapy, before the bubble bursts. For us, these questions include greater consideration of the role of community support structures, psychedelic therapy's long-term effects, what “safety” and “efficacy” mean amongst different stakeholder groups, and how best to set up harm reduction services and infrastructures, all before driving forward the mass scaling of pre-maturely-rigid formulations of psychedelic therapy. We recognize that this lies in tension with the imperative to scale, itself drawing on the language of a growing “mental health epidemic” and exacerbated by the Covid-19 pandemic. We maintain that by integrating the bubble's shadow before it bursts—or at least deflates [cf. (48)]—psychedelic therapy's potential to re-invigorate Western psychiatry might be better realized.

## Author Contributions

This manuscript emerged out of a series of conversations between TN and JM, followed by the joint writing of the abstract. TN led the writing of a first draft of the full manuscript. All authors then redrafted the manuscript, together and independently, and approved the submitted version.

## Conflict of Interest

The authors declare that the research was conducted in the absence of any commercial or financial relationships that could be construed as a potential conflict of interest.

## Publisher's Note

All claims expressed in this article are solely those of the authors and do not necessarily represent those of their affiliated organizations, or those of the publisher, the editors and the reviewers. Any product that may be evaluated in this article, or claim that may be made by its manufacturer, is not guaranteed or endorsed by the publisher.
